# Validity and Reliability Study of the Moodist Outcome Inventory (MOI)

**DOI:** 10.31083/AP49375

**Published:** 2025-12-23

**Authors:** Pelin Taş, Eslem Fulya Ekşi, Tuğçe Hilal Kadıoğlu, Kültegin Ögel

**Affiliations:** ^1^Department of Psychiatry, Moodist Hospital, 34660 İstanbul, Turkey; ^2^Department of Psychology, Moodist Hospital, 34660 İstanbul, Turkey

**Keywords:** follow-up, inventory, outcome, reliability, validity

## Abstract

**Background:**

The objective of this study is to develop an easily applicable scale to measure the course of treatment and the level of recovery for mental problems in various dimensions, which can be used in clinical practice and research.

**Methods:**

The validity and reliability test of Moodist Outcome Inventory (MOI) were conducted with 293 participants. Criterion-related validity was investigated by assessment with the Brief Psychiatric Rating Scale (BPRS), Disability Assessment Schedule (WHO-DAS-II), and Psychological Distress Scale (K10-PDS). Factor analysis was investigated by assessment with clinical and non-clinical samples. The sample was followed for six clinical assessments and evaluated by repetitive analysis of Variance (ANOVA) measurement.

**Results:**

The Cronbach’s alpha coefficient of the total scale was noted to be 0.89 in the reliability analysis. In the exploratory factor analysis, the single factor explaining 75.64% of the total variance was attained, and all items were included in this factor. Forty cases completed six clinical assessments, and the change between the MOI scores during the time interval was noted to be statistically significant. The correlation of the MOI scale with the K-10, WHO-DAS-II, and BPRS scales was noted to be 0.62, 0.73, and 0.65, respectively. In six consecutive assessments, the mean scores of all scales dropped significantly. The cut-off point of the scale was recorded as 7.27, and the reliable change index (RCI) was noted as 2.5.

**Conclusion:**

MOI was assessed as a valid and reliable scale for evaluating the course of treatment. The strengths of the scale are that it assesses both symptoms and well-being, is short, and can be implemented in clinical practice.

## Main Points

• Moodist Outcome Inventory (MOI) was assessed as a valid and reliable scale for evaluating the course of treatment.

• The strengths of the scale are that it evaluates both symptoms and well-being, it is short, the scoring system is uncomplicated, and it can be applied in clinical practice.

• MOI, which is developed as an objective treatment process assessment tool, is anticipated to contribute to research to be conducted in the field of mental health.

## 1. Introduction 

Are therapies a waste of time, or are they actually useful? Response to psychotherapies is quite variable, and it is difficult to predict which treatment option will be effective for which patient. Therefore, it is essential to identify the determinants that can ensure that the clinical course and response to therapy are tangible, replicable, and generalizable [1].

It is reported that clinical assessment of mental health should include the level of psychiatric symptoms, functionality, and patient satisfaction [2]. Positive outcomes of the treatment process imply a decrease in the individual’s symptoms and an increase in functionality and the general quality of life [3]. Nonetheless, alterations in the clinical course may not always be constant or in a linear order; accordingly, understanding the alterations throughout treatment is quite significant for recovery [4, 5, 6].

In order to evaluate the treatment process, scales assessing symptom level, functionality, or well-being are utilized [7, 8, 9, 10]. Yet, the number of scales evaluating the follow-up of the mental problem is quite limited in psychotherapy practices. One of the commonly utilized scales in this field is the Outcome Questionnaire (OQ-45.2) developed by Lambert *et al*. [11] in 1996. It is a 45-item scale based on self-report, evaluating three fundamental aspects of functioning: symptomatic functioning, social role, and interpersonal relationships. Despite OQ-45.2 being clinically appropriate for usage, it is criticized for being long and having a complicated scoring system [12].

Another commonly utilized scale to assess the course of psychotherapies is the Outcome Rating Scale (ORS) developed by Miller *et al*. in 2003 [13]. ORS was designed as an alternative to OQ-45, which was presumed to be long, focusing on four areas: individual, correlational, social functionality, and well-being. The limitations of the ORS include the inability to completely focus on the therapy process and assess the medical effect and the inability to provide adequate information regarding the nature of the individual’s symptoms [14].

The Brief Psychological Adjustment Scale (BASE-6) is another scale recommended for usage in assessing the clinical process. The purpose of this scale, which involves six items, is to provide more clinical information than ORS and to propose an alternative to the long OQ [12]. Even though the validity and reliability of the scale are statistically significant, the fact that it has not yet been adapted to other cultures or languages emerges as a limitation to its utilization [15].

Although the current evaluation tools are valid and reliable in the literature, limitations such as methodological complexity, inability to assess the treatment process entirely, duration of practice, and excessive cost make them challenging to apply in the clinical setting [16]. Hence, there is a necessity for practical scales to evaluate the clinical course in various aspects.

The objective of this study was to develop a scale to measure the course of treatment and the level of recovery for mental problems in various dimensions, which can be easily implemented in clinical practice and research, and to establish the validity and reliability of this scale.

## 2. Method

### 2.1 Development of the Scale

While creating the question pool, previously developed and used scale questions in this field were reviewed. It was decided to include four areas in the scale: well-being, mental state, relationships, and participation in life, which are among the factors suggested in previous studies on the outcome of therapy [2, 7, 11, 13].

Questions were created for each domain. Since it was aimed to design a short scale, similar questions for each domain were merged into one question. Two questions were selected for the mental state domain and one for the other domains.

A draft scale was applied to ten randomly selected cases. Ten people were deemed adequate because, based on our previous scale development experiences, we thought that sufficient information could be provided. For this procedure, patients who visited the outpatient clinic on the same day, who were free of psychosis, who were in adequate health to provide feedback, and who volunteered to participate were selected. Cognitive testing was conducted to assess the meaningfulness, clarity, and acceptability of successive versions of the test items of the Moodist Outcome Inventory (MOI). Items were revised after each round of interviews. To assess face and content validity, a panel of 10 experts was established to provide feedback on each item and the overall questionnaire.

The five questions developed were administered to 35 people. In the reliability analysis performed after the application, the corrected item-total correlation of one question in the mental state domain was 0.37. For the other questions, this value was above 0.7. Exploratory factor analysis was conducted using varimax rotation, and the factor loading of the same question was found to be below 0.3. Considering the cognitive review, expert opinions, and preliminary analysis results, this question was removed from the scale. The 4-item scale formed in line with the obtained information was named the MOI.

In the scale, in the field of well-being assessment, the items aim at exploring how the person feels in the process between two clinical assessments. The target is also to consider the quality of life in the assessment of well-being. For the mental state domain, it is investigated how the person evaluates their mental health in the process between two clinical assessments. The severity of the disease, i.e., the number of symptoms, was taken into consideration in the assessment of mental status.

The questions in the domains of relationships and participation in life were designed to assess the psychosocial functionality of the person. The participation in life domain was based on the person’s ability to work, education, fulfillment of household responsibilities, and self-care. If there is a significant deterioration in any of these areas or if there is a deterioration in more than one area, the option “poor” or “very poor” is marked.

The four questions created by this method are given in the attachment of the article. Each question is scored with a score between 0 and 4, and the lowest and highest scores are 0 and 16, respectively. A high score indicates a negative prognosis. The form was planned to be filled out by a clinical psychologist during the clinical assessment.

Two different forms were designed to assess the course of outpatients and inpatients. The question fields are similar and involve different assessments measuring the same variable. For instance, the question evaluating participation in life aims to evaluate the participation of outpatients in work, school, home, and fulfilling their responsibilities, whereas it aims to evaluate the participation of inpatients in-service activities. The question, which aims to assess the relationships, is based on the relationship with family, friends, etc. in the outpatient group, whereas it aims to determine the relationship of the individual in the inpatient group with other patients in the service and the therapy team. Two versions of the MOI scale are included in the attachment of the article.

MOI, unlike the scales used in outcome studies, assess many areas (number of symptoms, relationships, well-being and participation in life) that affect the outcome. On the other hand, its composition of four questions, simple scoring system, applicability to both inpatient and outpatient populations make it a practical scale in clinical practice.

This study was approved by Istanbul Istanbul Kent University Social Sciences and Humanities Research and Publication Ethics Committee (Approval Number: 13, Date: 5 January 2023).

### 2.2 Sampling

The clinical sampling consisted of patients who applied to a private psychiatric hospital for outpatient or inpatient treatment between 12 August 2023 and 30 October 2023, who had no communication issues, and from whom information could be obtained. In this study, a convenience sample was employed. The rationale for this choice lies in the development of a scale specific to the field of mental health. Since the scale was designed for a particular target group, the sample was drawn from patients who had applied to a hospital. Additionally, the scale was intended to serve as a monitoring tool. Although the present study was not a longitudinal one, the same sample was used during the follow-up phase to assess sensitivity to change—a key criterion for tools intended for monitoring purposes.

A hundred and forty-one individuals representing the clinical sampling were included in the research, 60 of whom were outpatients and 81 were inpatients. The non-clinical sampling was selected from hospital employees who had no active psychopathology, and the MOI scale was filled by 152 people in total. All participants received an informed consent form stating the details about the research, and participants who consented to volunteer approved this form.

The clinical sampling involved cases with various mental problems. The distribution of the cases based on mental problems is as follows: Anxiety disorder (n = 32, 22.7%), Major depression (n = 20, 14.2%), relationship issues (n = 16, 11.3%), psychosis (n = 10, 7.1%), bipolar (n = 8, 5.7%), psychotic bipolar (n = 2, 1.4%), alcohol use disorder (n = 20, 14.2%), substance use disorder (n = 38, 27.0%), and substance use-associated psychosis (n = 11, 7.8%). Since some cases have more than one mental problem, the percentages are different. Other characteristics of the sample are provided in the findings section.

### 2.3 Assessment Tools

Criterion-related validity was investigated by the Brief Psychiatric Rating Scale (BPRS), Disability Assessment Schedule (WHO-DAS-II), and Psychological Distress Scale (K10-PDS).

#### 2.3.1 Brief Psychiatric Rating Scale (BPRS)

The BPRS was developed in order to evaluate the severity of schizophrenia and other mental disorders and also provide information regarding anxiety, depression, thought disorders, aggression, and agitation [9]. This scale, consisting of 18 items, has a 6-point Likert-type scoring system. The Turkish validity and reliability study of the scale was carried out by Soykan in 1989 [17].

#### 2.3.2 Disability Assessment Schedule (WHO-DAS-II)

This scale, developed by the World Health Organization, was developed in order to assess the activity levels and social participation of the individual regardless of the medical diagnosis. The 5-point Likert-type scale consists of 12 items in total [10]. The Turkish validity and reliability study of the scale was performed by Uluğ *et al*. [18] in 2001. Examining the internal consistency of the scale, the Cronbach’s alpha coefficients of all subdomains were noted to range between 0.60 and 0.90. The Cronbach’s alpha value was found to be 0.92 for the overall scale.

#### 2.3.3 Psychological Distress Scale (K10-PDS)

It was developed to screen non-specific psychological distress and severe mental disorders [19]. The 10-item scale has a 5-point Likert-type scoring system. The Turkish validity and reliability study of the scale was carried out in 2019 by Altun *et al*. [20]. The internal consistency coefficient of the scale was noted to be 0.95.

### 2.4 Implementation

The forms used in the research were filled out by clinical psychologists who work at the hospital and have received training on this subject. A single assessment was conducted with the non-clinical sample group. The clinical sample group was planned to be administered six clinical assessments, and the interval between assessments was planned to be 7–15 days. In order to determine the change, MOI, K-10, WHO-DAS-II, and BPRS scales were applied in each clinical assessment. These six clinical assessments were conducted by the same clinical psychologist. Only on the day of the first assessment, a second clinical psychologist completed the MOI scale for the same participant. This was carried out to determine inter-rater reliability.

### 2.5 Statistical Analysis

As the mean education level of the sampling was high, the response options were classified as “below university” and “university”. Response options were categorized as “bad”, “good”, and “very good” in order to simplify the evaluation of economic status data.

Descriptive statistics are given as mean ± standard deviation since they are normally distributed, and categorical variables are given as frequency. The paired *t*-test was utilized to compare the mean scores of the first and sixth assessments. The Cronbach’s alpha coefficient was calculated for the reliability analysis of the MOI scale questions. Confirmatory and exploratory factor analyses were performed to reveal the construct validity of the scale. The two-way repetitive measurements ANOVA (Single Factor Repetitive) test was utilized for repetitive measurements. In cases where the sphericity assumption was not met, Greenhouse-Geisser correction was made.

The reliability of the change between clinical assessments was analyzed with the Reliable Change Index (RCI) method developed by Jacobson and Truax [21]. Comparison of clinical and non-clinical sampling and determination of the cut-off score were conducted via the cut-off score formula of Jacobson and Truax. A RCI is computed by dividing the difference between the pre-treatment and post-treatment scores by the standard error of the difference between the two scores. If the RCI is greater than the determined score, then the difference is reliable: a change of that magnitude would not be expected due to the unreliability of the measure. Conversely, if the RCI score is less than the determined score then the change is not considered to be reliable: it could have occurred just due to the unreliability of the measure.

All statistics in the research were performed using the SPSS 25.0 program (IBM Corp., Chicago, IL, USA).

## 3. Results

The sociodemographic characteristics of the clinical and non-clinical sampling are presented in Table 1.

**Table 1.** **The sociodemographic characteristics of the clinical and non-clinical sampling**.

		Clinical		Non-clinical		
		Mean	SD	Mean	SD	
Age		33.41	10.98	29.77	8.06	*p* = 0.003
		n	%	n	%	
Gender						
	Female	50	35.5	101	66.4	*p* < 0.001
	Male	91	64.5	51	33.6	
Marital status						
	Married	49	34.8	54	35.5	*p* = 0.890
	Other	92	65.2	98	64.5	
Educational level						
	Below University	72	51.1	51	33.6	*p* = 0.002
	University	69	48.9	101	66.4	
Economic Status						
	Good	81	57.4	63	41.4	*p *= 0.021
	Moderate	49	34.8	70	46.1	
	Poor	11	7.8	19	12.5	
Previous psychiatric treatment						
	None	32	22.7	110	72.4	*p* < 0.001
	Once	51	36.2	26	17.1	
	More than once	58	41.1	16	10.5	
Current medication use						
	None	64	45.4	138	90.8	*p* < 0.001
	Yes	77	54.6	14	9.2	

The Cronbach’s alpha coefficient of the entire scale was determined to be 0.89. The Cronbach’s alpha coefficient of the scale was found to be 0.9 in the outpatient clinical sampling and 0.89 in the inpatient clinical sampling. Table 2 shows the reliability coefficient of the MOI questions and how the Cronbach’s alpha coefficient of the scale is affected when an item is removed. In the follow-up assessments of the clinical sampling, the Cronbach’s alpha coefficient obtained in the second, fourth, and sixth assessments was determined to be 0.79, 0.85, and 0.84, respectively.

**Table 2.** **Reliability Coefficient of MOI Questions**.

	Scale average when an item is removed	Scale variance when an item is removed	Item-Total Correlation	Cronbach’s alpha coefficient of the scale when an item is removed
Well-being	5.13	7.98	0.77	0.86
Mental state	5.09	7.94	0.81	0.84
Relationships	5.28	7.97	0.73	0.87
Participation in life	5.34	8.00	0.74	0.87

The correlation between the clinical psychologists who made the assessments was 0.70 (*p* < 0.001) for well-being, 0.74 (*p* < 0.001) for mental state, 0.64 (*p* < 0.001) for relationships, 0.73 (*p* < 0.001) for participation in life, and 0.76 (*p* < 0.001) for the total scale score.

**Fig. 1.**
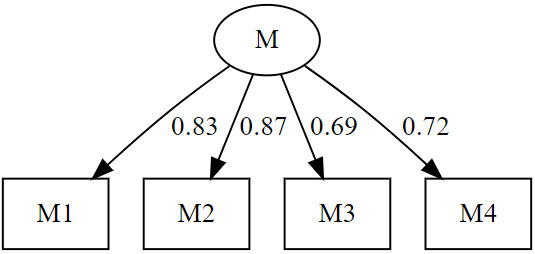
**MOI Scale Confirmatory Factor Analysis Graph**. MOI, moodist outcome inventory.

Exploratory factor analysis was conducted using varimax rotation with the main components method. Kaiser-Meyer-Olkin compatibility test value was noted to be 0.79, and the Bartlett Sphericity Test chi-square value was 340.819 (SD = 6, *p* < 0.001). The exploratory factor structure of MOI is presented in Table 3. In the exploratory factor analysis, a single factor with an eigenvalue greater than 1 was obtained, explaining 75.64% of the total variance. Similar findings were obtained in the outpatient and inpatient forms. All items were involved in a factor with factor loads greater than 0.30. The standardized factor loadings of the items forming the single dimension of the MOI Scale in the confirmatory factor analysis results can be seen in Fig. 1.

**Table 3.** **Exploratory factor structure of MOI**.

	Factor loads for the total scale	Factor loads for outpatient form	Factor loads for inpatient form
Well-being	0.90	0.92	0.88
Mental State	0.89	0.91	0.88
Relationships	0.84	0.85	0.85
Participation in life	0.84	0.84	0.85

The fit indices (goodness-of-fit indices and adjusted chi-square (χ^2^/df) value) for the dimensions included in the model established to test the confirmatory factor analysis can be seen in Table 4. When examining the model results; the RMSEA fit index is 0.055, indicating an acceptable fit. Other fit indices, including NFI, NNFI, CFI, IFI, RFI, SRMR, GFI, and AGFI, also demonstrate an acceptable fit to a good fit. Accordingly, the acceptability of the fit indices, along with the adjusted chi-square value showing a good fit, suggests that our data exhibit an acceptable fit and that our model is statistically significant and valid (*p* = 0.001; *p* < 0.01).

**Table 4.** **MOI scale confirmatory factor analysis fit indices**.

Fit indices	Good fit	Acceptable fit	Results of model	Fit
RMSEA	0 < RMSEA < 0.05	0.05 ≤ RMSEA ≤ 0.10	0.055	Acceptable
NFI	0.95 ≤ NFI ≤ 1	0.90 ≤ NFI ≤ 0.95	0.963	Good fit
NNFI	0.97 ≤ NNF ≤ 1	0.95 ≤ NNFI ≤ 0.97	0.952	Acceptable
CFI	0.97 ≤ CFI ≤ 1	0.95 ≤ CFI ≤ 0.97	0.974	Good fit
IFI	0.95 ≤ IFI ≤ 1	0.90 ≤ IFI ≤ 0.95	0.975	Good fit
RFI	0.90 ≤ RFI ≤ 1	0.85 ≤ RFI ≤ 0.90	0.888	Acceptable
SRMR	0 ≤ SRMR ≤ 0.05	0.05 ≤ SRMR ≤ 0.10	0.038	Good fit
GFI	0.95 ≤ GFI ≤ 1	0.90 ≤ GFI ≤ 0.95	0.965	Good fit
AGFI	0.90 ≤ AGFI ≤ 1	0.85 ≤ AGFI ≤ 0.90	0.857	Acceptable
χ^2^/df *(6124/2)*	0 ≤ χ^2^/df ≤ 3	3 ≤ χ^2^/df ≤ 5	3062	Acceptable

RMSEA, root mean square error of approximation; NFI, normed fit index; NNFI, non-normed fit index; CFI, comparative fit index; IFI, incremental fit index; RFI, relative fit index; SRMR, standardized root mean square residual; GFI, goodness of fit index; AGFI, adjusted goodness of fit index; χ^2^/df, chi-square/degrees of freedom.

MOI was first administered to 293 cases, of whom 40 cases completed six assessments. The mean number of days between the first and second assessments was found to be 7.49 ± 6.15, the mean number of days between the second and third assessments was 6.45 ± 3.72, the mean number of days between the third and fourth assessments was 8.05 ± 5.29, the mean number of days between the fourth and fifth assessments was 7.50 ± 4.31, and the mean number of days between the fifth and sixth assessments was 9.34 ± 6.75.

**Fig. 2.**
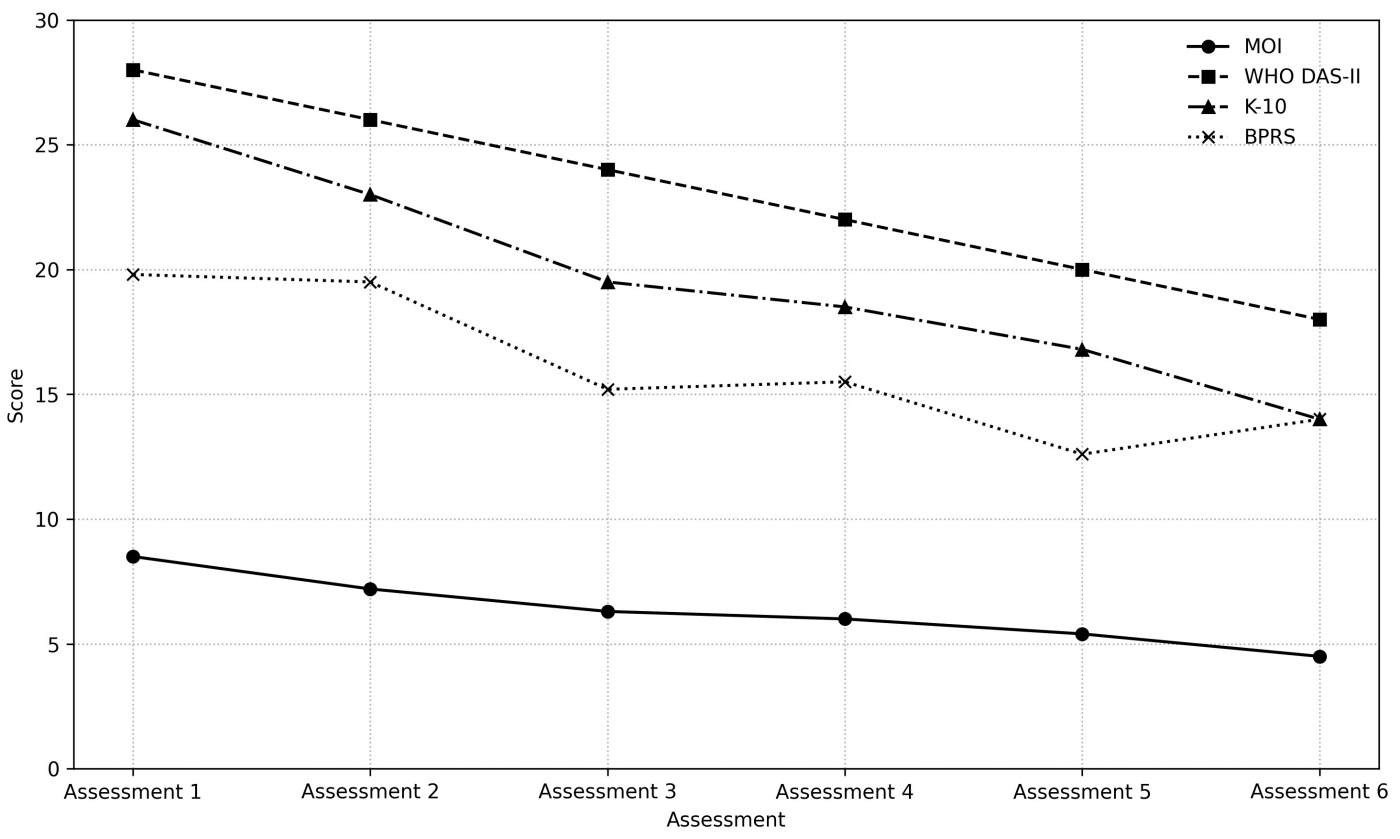
**The mean scores of MOI, K-10, WHO-DAS-II, and BPRS scales in six clinical assessments**. WHO-DAS-II, Disability Assessment Schedule; BPRS, Brief Psychiatric Rating Scale; K-10, Psychological Distress Scale.

The correlation of the MOI scale with K-10, WHO-DAS-II, and BPRS scales was noted to be strong and statistically significant (Table 5). It is noteworthy that the mean scores of all scales decreased significantly in six assessments (Fig. 2). The mean MOI scale score implemented in the first assessment was 8.56 ± 3.94 and the mean score in the sixth assessment was 4.42 ± 2.90. The difference between the mean scores of the first and sixth assessments was statistically significant (t = 7.06; SD = 39; *p* < 0.001). In the analysis conducted with repetitive ANOVA measurement with Greenhouse-Geisser correction, the change between MOI scores throughout the time interval was detected to be statistically significant (F = 27.25; SD = 2.94; *p* < 0.001).

**Table 5.** **The mean score of the MOI, K-10, WHO-DAS-II, and BPRS scales according to clinical assessments and the correlation between the MOI scale and the other scales**.

	MOI			K-10				WHO-DAS-II				BPRS			
	n	Mean	SD	n	Mean	SD	Cor.	n	Mean	SD	Cor.	n	Mean	SD	Cor.
Assessment 1	141	8.56	3.94	80	26.33	10.96	0.62	130	28.03	12.08	0.73	53	19.81	22.35	0.65
Assessment 2	114	7.32	4.04	67	23.16	9.98	0.61	106	26.32	12.27	0.73	45	19.51	21.22	0.74
Assessment 3	92	6.35	3.59	53	19.49	7.70	0.68	88	24.00	11.49	0.74	37	15.10	14.78	0.73
Assessment 4	68	5.98	3.93	42	18.45	7.49	0.65	65	22.60	11.01	0.78	22	15.40	20.42	0.69
Assessment 5	55	5.45	3.25	38	16.94	8.34	0.71	55	20.60	9.16	0.79	16	12.75	19.35	0.70
Assessment 6	40	4.42	2.91	27	14.00	6.40	0.61	40	18.25	8.62	0.73	11	16.36	22.21	0.64

Cor, correlation; *p* < 0.001 in all correlations.

The mean total score of the MOI scale was 8.56 ± 3.94 in the clinical sample and 5.40 ± 2.67 in the non-clinical sample. The mean MOI score is the average of the total scores of the MOI administered at the first evaluation. The difference between the two averages is statistically significant (t = –7.97; df = 286; *p* < 0.001).

The retention rate and participation in all six interviews in the study were very low (21.97%) among the clinical sample. On the other hand, there was no significant difference in the mean age between those who completed the study (32.03 ± 11.09) and those who did not (30.83 ± 9.30) (t-value: –2.57; *p* = 0.11). Similarly, no statistically significant differences were observed between participants who completed the study and those who did not in terms of gender (χ^2^ = 1.93, *p* = 0.16), marital status (χ^2^ = 0.01, *p* = 0.93), education level (χ^2^ = 5.08, *p* = 0.27), economic status (χ^2^ = 6.57, *p* = 0.16), previous treatments (χ^2^ = 2.66, *p* = 0.24), or number of children (χ^2^ = 0.53, *p* = 0.46). No significant difference was found between individuals with substance use disorders and those with general psychiatric disorders (χ^2^ = 2.16, *p* = 0.14). The completion rate among inpatients (14.8%) was found to be significantly lower than that of outpatients (46.7%) (χ^2^ = 17.2, *p* < 0.001).

In the calculation performed via the formula developed by Jacobson and Truax, the cut-off point of the scale was found to be 7.27, and the RCI to be 2.5. In other words, a 2.5-point decrease from the total score of the scale indicates a reliable alteration.

## 4. Discussion

In this research, the validity and reliability of the MOI scale were examined. The obtained psychometric data revealed that the scale was valid and reliable. Although it is a very short scale, its internal consistency was noted to be quite high.

As one of the factors determining the level of recovery, a decrease in the number of symptoms of the patient was reported [22]. In this research, K-10 PDS and BPRS scales were utilized in order to measure the symptoms, that is, the psychopathology level of the patients. It was seen that the correlation between the developed MOI scale and the said scales was statistically significant. It was also detected that K-10 PDS, BPRS, and MOI scales demonstrated a similar trend in the process. Hence, we can state that the MOI scale measures the level of psychopathology.

It is also known that another factor that determines the level of recovery is functionality [23]. For this purpose, the WHO-DAS-II scale and MOI were compared in the research. In the first and subsequent assessments, it was established that the MOI scores correlated with the WHO-DAS-II scale scores. We may consider these findings an indication that MOI assesses functionality.

It has been stated that monitoring scales should be sensitive to change [24]. In this study, it was determined that the total score of the MOI scale displayed a change in the subsequent stages of the assessments, and this change was statistically significant. In line with these findings, it might be suggested that MOI is sensitive to change. On the other hand, it is not possible to claim with this study whether these results stem from treatment effectiveness or confounding factors.

Comparing the clinical sampling scores of MOI with the non-clinical sampling, it was observed that the difference between the mean MOI total score of the two groups was significant. Hence, we may suggest that the scale has a discriminating feature. Distinguishing the clinical and nonclinical sampling and comparing their scores will also provide information regarding the quantitative level of recovery in treatment. The individual may display a certain rate of recovery; however, how close they will reach the level of non-clinical sampling can be decided with these data [21]. If six interviews had also been conducted for the non-clinical sample and the mean scores of the clinical and non-clinical sample groups had been compared, the performance of the MOI scale could have been better evaluated. We believe that conducting such a study could be beneficial for assessing the scale’s performance.

It is remarkable that the scale has a similar factor structure and internal consistency in outpatients and inpatients. Some scales have been determined to indicate divergent psychometric characteristics for inpatients or outpatients [25]. Nonetheless, no such difference was noted in the scale we developed, and as such, we believe that the scale may be utilized in both inpatients and outpatients.

In various mental disorders, the course of the disease seems to be different [26]. Being aware of the changes to be observed throughout the course of various mental disorders is a tool to assist the clinician. Thus, measuring the efficacy of MOI in different disorders will enhance the strength of the scale.

Addiction is a multifactorial issue. Family, social support, work and education life, biological factors, etc. play a role. People who used alcohol and substances were also involved in the research sampling, and the MOI scale was detected to be valid and reliable in the analyses. However, as there are many and various factors determining the course of addiction, we believe that it would be more practical to utilize scales such as the Addiction Outcome Assessment Index (AOAI) to monitor the process of alcohol and substance use [27].

## 5. Limitations

There are some limitations in our research. The first limitation is that the non-clinical sample is predominantly female and the clinical sample is predominantly male. Since the proportion of substance users in the study is high, the clinical sample has been predominantly male. This may have a lower impact on the validity and reliability of the scale; however, it should not be overlooked that gender is an important factor in outcomes and that gender could influence mental health outcomes. Significant differences were found between the clinical and non-clinical groups in several sociodemographic variables, including age, educational level, history of psychiatric treatment, and current medication use. These discrepancies may limit the normative representativeness of the groups in establishing the cut-off point. Therefore, the findings should be interpreted as specific to the present sample, and caution is warranted when attempting to generalize the results to broader populations. Another limitation of the study was, economic status was assessed via subjective categories (“bad”, “good”, “very good”) rather than objective indicators (e.g., income, occupation). This may introduce potential bias and limits interpretability.

This study employed a convenience sampling method, which may limit the generalizability of the findings to broader populations. As such, the results should be interpreted with caution and considered applicable primarily to groups with similar characteristics. To establish the robustness of the scale’s psychometric properties, further validation is recommended across diverse samples, including variations in age, gender, and socioeconomic status.

The retention rate and participation in all six interviews in the study were found to be low. This may influence the total MOI scores and their correlations with other scales. Small subsamples in correlation analyses may make the results less stable over time. Previous studies have reported high dropout rates in psychiatric clinics (45%) [28]. Low financial protection has been identified as one of the reasons for this. Since this study was conducted in a private clinic, this dropout rate is considered acceptable. On the other hand, when comparing those who completed the study with those who did not, no significant differences were found between the two groups across a range of variables. Given that this study is a validity and reliability study of an outcome measure, multiple imputation was not considered. It should nevertheless be kept in mind that a high drop-out rate may reduce the generalizability of the findings. We believe that in future outcome studies using this scale, attention should be paid to keeping dropout rates low.

Another limitation is that the time between clinical assessments varied between participants. This may cause difficulties in interpreting the results. In the sampling of the research, it was observed that the educational level was high. It has been demonstrated in some previous studies that the level of education may make a difference in answering the forms [29]. Since the MOI is planned as a scale to be applied by clinicians, it can be said that the level of education will not be very effective. Studies should be conducted in different educational levels and genders, to eliminate these limitations and for the generalizability of the scale.

## 6. Conclusion

In light of these findings, it may be suggested that the MOI is a valid and reliable scale in the evaluation of the treatment process of various mental illnesses. It has the potential to provide advantages in clinical practice because it evaluates the individual in terms of symptoms, well-being, and participation in life. Moreover, it is short, has an uncomplicated scoring system, and is sensitive to change. It is predicted that the MOI developed as an objective treatment process assessment tool will contribute to research in the field of mental health.

## Availability of Data and Materials

The data supporting the findings of this study are not publicly available due to privacy and ethical constraints. Nevertheless, they can be obtained from the corresponding author upon reasonable request and with the approval of Moodist Hospital.

## Appendix

**Attachment 1: Moodist Outcome Inventory (MOI)**

**Moodist Outcome Inventory -MOI**

**Table:**

Ask the questions directly to the person and mark the answer. If the person is not able to identify their own condition (e.g., psychosis, manic episode, etc.), then the form should be completed by clinicians. Do not include impaired functionality due to physical (or environmental) constraints.

**1. Well-being **

How did you feel during the last week (when we were not in contact)?

0-Very good 1-Good 2-Average 3-Bad 4-Very bad

*If the person is not in a position to determine their own condition (e.g., psychosis, manic episode, etc.), then well-being and quality of life should be assessed by the clinician.*

**2. Mental state**

How would you assess your mental health during the last week (when we were not in contact)?

0-Very good 1-Good 2-Average 3-Bad 4-Very bad

*If the person is not in a position to determine their mental health (e.g., psychosis, manic episodes, etc.), then the clinician should assess the severity of the illness, i.e. the number of symptoms. *

**Table:**

In the following questions; if there is significant deterioration in any of the living spaces or deterioration in more than one space; mark it as “bad” or “very bad.”

**3. Relationships**

During the last week (when we were not in contact), how were your relationships with your family and/or close friends?

0-Very good 1-Good 2-Average 3-Bad 4-Very bad

**4. Participation in life - taking responsibility for work, school, home, and self**

During the last week (when we were not in contact); was your working or educational life negatively affected OR did you have difficulty in carrying out your household responsibilities (e.g., cleaning, cooking, childcare, laundry, etc.) OR did you have difficulty in taking care of yourself (e.g., bathing, dressing, eating, body cleaning, etc.)?

0-Very good 1-Good 2-Average 3-Bad 4-Very bad

**Moodist Outcome Inventory Inpatient Form-MOI-I**

**Table:**

Ask the questions directly to the person and mark the answer. If the person is not able to identify their own condition (e.g., psychosis, manic episode, etc.), then the form should be completed by the clinicians. Do not include impaired functionality due to physical (or environmental) constraints.

**1. Well-being**

Ask him/her how he/she felt during the last week (when we were not in contact).

0-Very good 1-Good 2-Average 3-Bad 4-Very bad

*If the person is not in a position to determine their own condition (e.g., psychosis, manic episode, etc.), then well-being and quality of life should be assessed by the clinician. *

**2. Mental state**

Answer according to the severity of the disease and/or the number of symptoms lately.

0-Very little/None   1-Less   2-Average   3-More   4-Too much

*If the person is not able to determine their own condition (e.g., psychosis, manic episode, etc.), then the severity of the illness and/or the number of symptoms should be assessed by the clinician. *

**Table:**

In the following questions; if there is significant deterioration in any of the living spaces or deterioration in more than one space; mark it as “bad” or “very bad.”

**3. Relationships**

How have their relationships with family, friends, or service staff been lately?

0-Very good 1-Good 2-Average 3-Bad 4-Very bad

**4. Participation in life - taking responsibility for work, school, home, and self**

What is the level of their participation in activities in the ward, leaving their room, communication with other ward patients, or self-care (cleaning, dressing, makeup, etc.) lately?

0-Very good 1-Good 2-Average 3-Bad 4-Very bad
